# Molecular Evolution, Diversity, and Adaptation of Influenza A(H7N9) Viruses in China

**DOI:** 10.3201/eid2410.171063

**Published:** 2018-10

**Authors:** Jing Lu, Jayna Raghwani, Rhys Pryce, Thomas A. Bowden, Julien Thézé, Shanqian Huang, Yingchao Song, Lirong Zou, Lijun Liang, Ru Bai, Yi Jing, Pingping Zhou, Min Kang, Lina Yi, Jie Wu, Oliver G. Pybus, Changwen Ke

**Affiliations:** Guangdong Provincial Center for Disease Control and Prevention, Guangzhou, China (J. Lu, Y. Song, L. Zou, L. Liang, R. Bai, Y. Jing, P. Zhou, M. Kang, L. Yi, J. Wu, C. Ke);; Guangdong Provincial Institution of Public Health, Guangzhou (J. Lu, P. Zhou, L. Yi);; University of Oxford, Oxford, UK (J. Raghwani, R. Pryce, T.A. Bowden, J. Thézé, O.G. Pybus);; Beijing Normal University, Beijing, China (S. Huang)

**Keywords:** avian influenza, avian influenza virus, viruses, influenza, H7N9, virus subtypes, phylogenetics, molecular evolution, molecular epidemiology, adaptation, diversity, respiratory infections, hemagglutination inhibition assay, China

## Abstract

The substantial increase in prevalence and emergence of antigenically divergent or highly pathogenic influenza A(H7N9) viruses during 2016–17 raises concerns about the epizootic potential of these viruses. We investigated the evolution and adaptation of H7N9 viruses by analyzing available data and newly generated virus sequences isolated in Guangdong Province, China, during 2015–2017. Phylogenetic analyses showed that circulating H7N9 viruses belong to distinct lineages with differing spatial distributions. Hemagglutination inhibition assays performed on serum samples from patients infected with these viruses identified 3 antigenic clusters for 16 strains of different virus lineages. We used ancestral sequence reconstruction to identify parallel amino acid changes on multiple separate lineages. We inferred that mutations in hemagglutinin occur primarily at sites involved in receptor recognition or antigenicity. Our results indicate that highly pathogenic strains likely emerged from viruses circulating in eastern Guangdong Province during March 2016 and are associated with a high rate of adaptive molecular evolution.

Since its first detection in March 2013, avian influenza A(H7N9) virus has caused 1,534 human infections that, as of November 30, 2017, had resulted in 608 deaths. Recurrent waves of human cases have been reported in 27 provinces in China, indicating sustained transmission of H7N9 viruses (*1*). Moreover, since its emergence, H7N9 virus has reassorted with influenza A(H9N2) viruses that co-circulate in China, resulting in an increasingly diverse array of virus genomes (*2*–*4*). The fifth influenza epidemic wave (2016–17) was marked by a notable increase in the number of human cases (677 during September 2016–May 2017), making it the largest outbreak of influenza A(H7N9) since 2013. Moreover, geographic distribution of human cases suggests that H7N9 virus is now more widespread and that residences of patients have shifted gradually from urban to semiurban and rural areas (*1*,*5*–*7*). These epidemiologic observations have raised public health concerns.

Previous molecular surveillance studies suggested that H7N9 virus lineages originate in 2 densely populated areas, the Yangtze River Delta region in eastern China and the Pearl River Delta region in central Guangdong Province (*8*). Preliminary epidemiologic data suggested that most human infections in the current fifth epidemic wave were caused by viruses from the Yangtze River Delta region (*5*) (previously named lineage C viruses) (*3*). These viruses, in contrast to viruses from the Pearl River Delta region (previously named lineage B viruses) (*3*), appear to exhibit reduced cross-reactivity with existing candidate vaccine virus strains (*9*). Furthermore, a subset of lineage C isolates has also acquired a highly pathogenic (HP) phenotype (*5*,*10*,*11*).

These observations suggest that the increased epidemic activity of H7N9 viruses in China might be driven, at least in part, by ongoing virus evolution and adaptation. Decreased cross-reactivity and increased pathogenicity of some H7N9 viruses was discovered only recently (*9*), and the genetic diversity and evolution of the current fifth epidemic wave of these viruses are not yet well understood. Information necessary to clarify this situation includes geographic distribution of currently circulating H7N9 virus lineages, origin and genetic composition of newly emerged HP H7N9 viruses, and evolutionary and structural characterization of mutations associated with the fifth epidemic wave of these viruses.

We report 47 hemaglutinnin (HA) and 43 neuraminidase (NA) gene sequences of human-derived and poultry-derived H7N9 viruses that were isolated during 2015–2017 in Guangdong Province, China. We conducted structural and evolutionary analyses of these strains and characterized the evolution and emergence of currently circulating H7N9 viruses in China.

## Materials and Methods

### Ethics

This study was approved by the institutional ethics committee of the Center for Disease Control and Prevention of Guangdong Province. Written consent was obtained from patients or their guardian(s) when samples were collected. Patients were informed about the study before providing written consent, and data were anonymized for analysis.

### Sample Collection

Samples from persons with suspected cases of influenza A(H7N9) were initially tested for avian influenza A virus in provincial clinics in Guangdong Province. Specimens with positive results were subsequently analyzed (*12*,*13*). For poultry-related samples, we obtained samples from locations where poultry were housed and processed (e.g., cages, feeding troughs, defeathering machines) (*12*). Respiratory specimens were collected from persons with suspected cases of influenza A(H7N9) by the Ministry of Health of China.

### Sequence Alignment

For phylogenetic studies, we sequenced 47 HA and 41 NA sequences from 20 human samples and 28 poultry-related samples; all belonged to the fourth and fifth epidemic waves of influenza A(H7N9) (GISAID [https://www.gisaid.org/] accession nos. EPI866538–77, 972231–6, 972238–303, 974029, 974523, 974539–42, 997159–60, and 1171786–93). These new H7N9 sequences were combined with all available H7N9 gene sequences whose sampling dates and locations were known. Two gene sequence datasets were generated: H7, HA (n = 737) and N9, NA (n = 610). We constructed multiple sequence alignments by using ClustalW (*14*) and edited these sequences manually by using AliView (*15*).

### Molecular Clock Phylogenetic Analysis

We estimated molecular clock phylogenies by using the Bayesian Markov Chain Monte Carlo approach implemented in BEAST version 1.8 (*16*) as described (*4*). We computed 4 independent Markov Chain Monte Carlo runs of 1.5 × 10^8^ steps for each alignment and extracted a subset of 2,000 phylogenies from the posterior tree distribution, subsequently used as an empirical tree distribution for phylogeographic analyses (*17*). We computed maximum clade credibility trees for each dataset by using TreeAnnotator (*16*).

### Phylogeographic Analysis of Influenza A(H7N9) Epidemic

We used the discrete phylogeographic method (*18*) implemented in BEAST to investigate spatial dynamics of H7N9 virus lineages from 6 regions in China as classified in a previous study (*4*). The 6 locations were eastern China (Anhui, Shanghai, Zhejiang, Jiangsu, and Shandong); central China (Jiangxi and Hunan); northern China (Beijing, Henan, Hebei, and Xinjiang); southeastern China (Fujian); central Guangdong Province (Guangzhou, Huizhou, Foshan, Dongguan, Zhongshan, Shenzhen, Jiangmen, Zhaoqing Yangjiang, Maoming, and Yunfu); and eastern Guangdong Province (Meizhou, Heyuan, Chaozhou, Jieyang, Shantou, Shanwei, and Shaoguan).

Because sporadic human cases detected in Malaysia and Taiwan were believed to have originated in China, we used available epidemiologic information to assign their location to the most likely source in China. Hong Kong and central Guangdong Province were treated as a single location because of their proximity to each other. We analyzed reported H7N9 virus infections and virus sequences (Technical Appendix Table 1). To estimate directionality of virus lineage movement, we used asymmetric continuous-time Markov chain phylogeographic model (*19*) and a Bayesian stochastic search variable selection procedure (*18*).

### Inferring Phylogenetic Distribution of Amino Acid Changes

We investigated phylogenetic positions of amino acid changes among H7N9 virus isolates by using HA and NA maximum clade credibility trees. We estimated maximum posterior probability amino acid sequences for each internal node by using BEAST with a Jones–Taylor–Thornton amino acid substitution model (*20*), gamma-distributed among-site rate heterogeneity (*21*), and a strict molecular clock model. To infer amino acid substitutions along the trunk branches of the H7N9 phylogeny, we mapped amino acid changes onto internal branches by using a Java script (available on request). Trunk branches corresponded to internal branches that subtended >5 terminal nodes in the fifth influenza epidemic wave.

### Structure-Based Mapping Analysis

We used the crystal structure of the HA (Protein Data Bank no. 4BSE) (*22*) and NA (Protein Data Bank no. 2C4L) glycoproteins from an influenza A(H7N9) virus to map amino acid changes identified by evolutionary analysis. We performed residue mapping onto the H7 and N9 structures by using PyMol (*23*). We calculated solvent accessibility for trimeric hemagglutinin with the ligands removed by using ESPript (*24*) and identified receptor-binding residues by using CONTACT in CCP4 (*25*).

### Positive Selection Analyses

To identify sites under positive selection, we used methods implemented in HyPhy (*26*) to estimate the dN/dS ratio of codons in HA. These methods included single-likelihood ancestor counting (*27*), fixed effects likelihood (*27*), mixed effects model of evolution (*28*), and the fast unconstrained Bayesian approximation approach (*29*).

### Estimating Rates of Virus Molecular Adaptation

We estimated rates of adaptive substitution in H7N9 virus HA and NA genes by using an established population genetic method related to the McDonald-Kreitman test (*30*,*31*). We used a consensus of H7N9 first-wave sequences as an outgroup to estimate derived and ancestral mutational site frequencies in each subsequent wave. Specifically, we classified polymorphisms into 3 categories according to their frequency in the population (low, 0%–15%; intermediate, 15%–75%; and high, 75%–100%). We calculated the number of adaptive substitutions from the number of synonymous and nonsynonymous sites in each category and assessed statistical uncertainty by using a bootstrap approach (1,000 replicates) (*30**,**31*).

### Serologic Analysis

We obtained serum samples from 4 patients with influenza A(H7N9) 2–3 weeks after clinical symptoms were observed. We performed hemagglutination inhibition assays by using different lineages of H7N9 viruses as antigens (online Technical Appendix). Three lineage C1 strains, 4 lineage C2 strains, 5 lineage B strains, and 4 HP strains were used as antigens (Table). We calculated serum titer for each H7N9 strain as the highest reciprocal serum dilution providing complete hemagglutination inhibition.

**Table:** Hemagglutination inhibition tilters of serum from 4 patients infected with influenza A(H7N9) virus against other influenza viruses, China*

H7N9 strain	Date of collection	Clade	Titer by patient and H7N9 strain			
			P1, strain NA	P2, strain NA	P3, strain ZS29	P4, strain ST18
EPI972232/A/CZ009†	2017 Jan	C1	2,048	2,048	512	512
EPI972243/A/MZ011†	2017 Jan	C1	4,096	4,096	1,024	512
EPI1171792/A/ST18†	2017 Jan	C1	1,024	512	512	512
EPI656434/A/ST72	2015 Feb	C2	256	128	1024	128
EPI866569/A/ST021†	2016 Feb	C2	256	128	512	128
EPI656314/A/SW20	2015 Jan	C2	256	128	512	128
EPI1171791/A/SW33†	2015 Feb	C2	256	128	512	128
EPI972259/A/ZS201†	2016 Dec	B	256	128	512	128
EPI972234/A/FS10†	2017 Jan	B	256	256	512	128
EPI656054/A/ZS74	2014 Jan	B	512	256	512	128
EPI656038/A/ZS71	2014 Jan	B	256	256	512	128
EPI656014/A/GZ66	2014 Jan	B	512	256	512	64
EPI1171786/Env/YJ370†	2017 Sep	HP	64	64	512	64
EPI1171788/Env/YJ073†	2017 May	HP	64	64	512	64
EPI1171790/A/ZS29†	2017 Mar	HP	128	64	512	64
EPI919607/A/17SF003	2017 Jan	HP	256	64	512	128

*HP, highly pathogenic; NA, not available; P, patient no. †Strains isolated and sequenced in this study.

## Results

### Molecular Epidemiology of Viruses Isolated during 2013–2017

**Figure 1:**
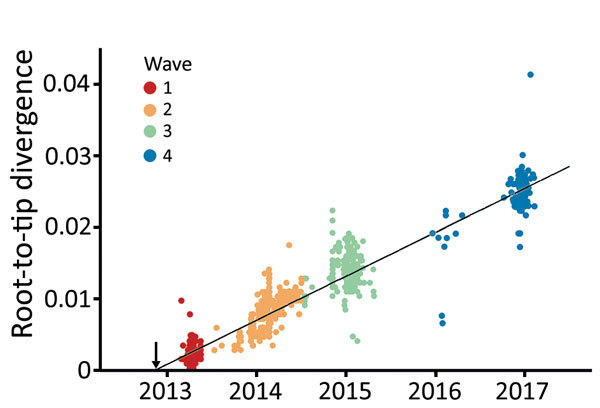
Regression of root-to-tip divergence estimated from hemagglutinin gene of influenza A(H7N9) viruses, China. Arrow indicates the time of the most recent common ancestor of the epidemic lineage.

**Figure 2:**
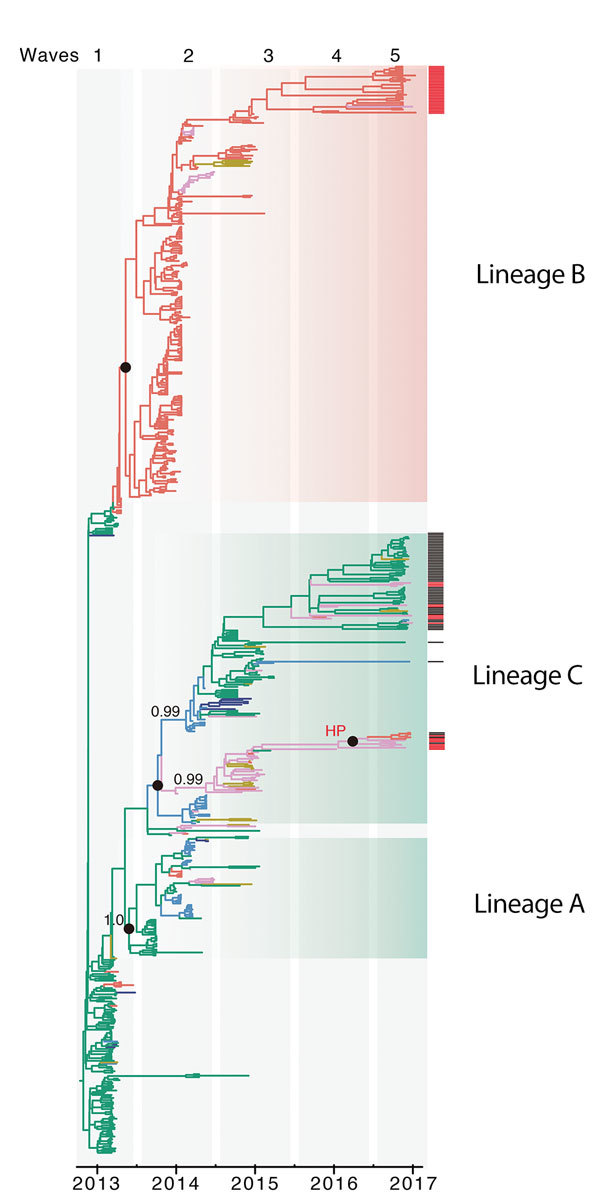
Genetic evolution and spatial spread of epidemic lineage of influenza A(H7N9) viruses, China, 2013–2017. Bayesian maximum clade credibility tree of the hemagglutinin gene is shown. Black bars to the right of the tree indicate sequences (from waves 4 and 5) from other studies (*1*,*5*), and red bars indicate sequences reported in this study from Guangdong Province. Branch colors indicate most probable ancestral locations of each branch. Three major lineages (A, B, and C) of H7N9 viruses were observed. Values along branches indicate bootstrap values. Black circles indicate posterior support >0.95. Location of posterior support is shown for selected clades. An H7N9 strain closely related to the highly pathogenic H7N9 virus cluster is indicated. HP, highly pathogenic.

During 2013–2017, the influenza A(H7N9) virus epidemic lineage was geographically structured and classified into 3 major lineages, A, B, and C, in accordance with the lineage naming scheme used in a previous study (*3*). H7N9 virus has evolved in a clock-like manner (i.e., there is a strong linear relationship between genetic divergence and sampling time; correlation coefficient 0.95) (Figure 1). The estimated time to the most recent common ancestor (TMRCA) of H7N9 virus HA sequences is November 2012 (95% credible region October–December 2012). The corresponding molecular clock phylogeny for NA (Technical Appendix Figure 1) also shows A–C lineages and has a similar estimated TMRCA of September 2012 (95% credible region July–October 2012). However, the topology of the NA phylogeny differs from that of HA, suggesting reassortment between HA and NA during emergence of the H7N9 virus epidemic lineage (Figure 2; Technical Appendix Figure 1).

**Figure 3:**
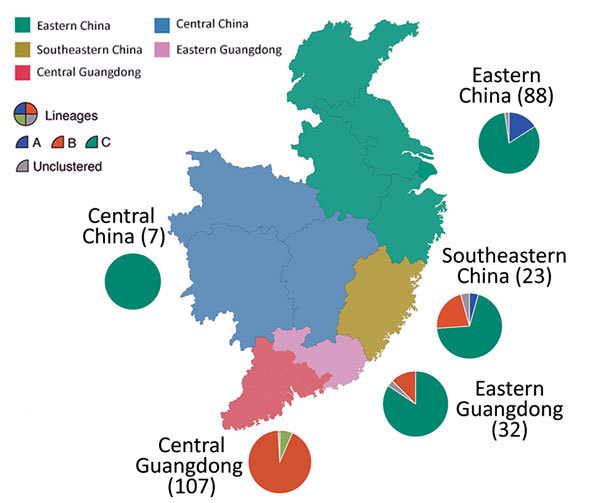
Geographic location and lineage classification of 374 influenza A(H7N9) human viruses, China. Values in parentheses indicate number of sequenced viruses from each region. Pie charts indicate approximate percentages of each virus lineage (A, B, C, or unclustered). Sequences from Xinjiang Province in northern China are not shown.

Different H7N9 virus lineages are associated with different epidemiologic patterns (Figures 2, 3). Specifically, most (86%, 32/37) lineage B viruses that were isolated during the fourth and fifth influenza epidemic waves descended from viruses circulating in central Guangdong Province during earlier epidemic seasons (Figure 2). In addition, lineage B viruses isolated from the fourth and fifth influenza waves were almost exclusively restricted to central (rather than eastern) Guangdong Province (Figures 2, 3). In contrast, viruses in eastern China, composed of 2 lineages (A and C) have been exported to and become dominant in multiple regions as the epidemic has progressed (*3*). These findings indicate a comparatively broader geographic dissemination (Figure 3; Technical Appendix Figure 1).

**Figure 4:**
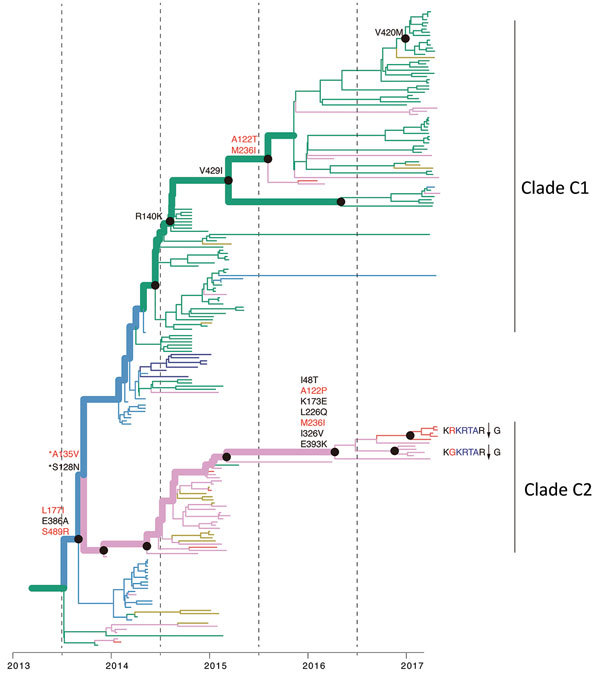
Reconstruction of amino acid changes along trunk of lineage B phylogenies of influenza A(H7N9) viruses, China. Maximum clade credibility tree of hemagglutinin gene sequences from lineage B is shown. Branches are colored according to geographic locations, as in Figure 3. Thicker lines indicate the trunk lineage leading up to the current fifth influenza epidemic wave. Amino acid changes along the trunk are indicated. Red branches indicate sites undergoing parallel amino acid changes across multiple lineages. Mutations correspond to H3 numbering scheme. *Indicates uncertainty about the phylogenetic position of the A135V and S128N mutations because branch posterior support is low.

The new isolates from eastern Guangdong Province, combined with isolates from eastern China (*1*,*5*), suggest that recent H7N9 virus activity is driven primarily by lineage C viruses (Figure 2). The estimated TMRCA of lineage C is December 2013 (95% highest posterior density October 2013–January 2014). For lineage C, we observed 2 clades (Figure 4). The larger of these clades (C1) circulates mainly in central and eastern China, and the smaller clade (C2) is found predominantly in eastern Guangdong Province. Clade C2 also includes recently identified HP viruses (Figures 1, 2, 4).

To investigate these HP viruses, we undertook retrospective screening of poultry-related samples collected in Guangdong Province during January 2016–February 2017 and identified 7 HP influenza virus isolates that belong to the HP cluster within C2 (Figure 2). These HP viruses also form a distinct cluster within lineage C viruses in the NA phylogeny Technical Appendix Figure 1). Our analyses indicated that the HP clade likely emerged from clade C2 viruses circulating in eastern Guangdong Province in 2016.

### Adaptive Evolution in Virus C Lineage

**Figure 5:**
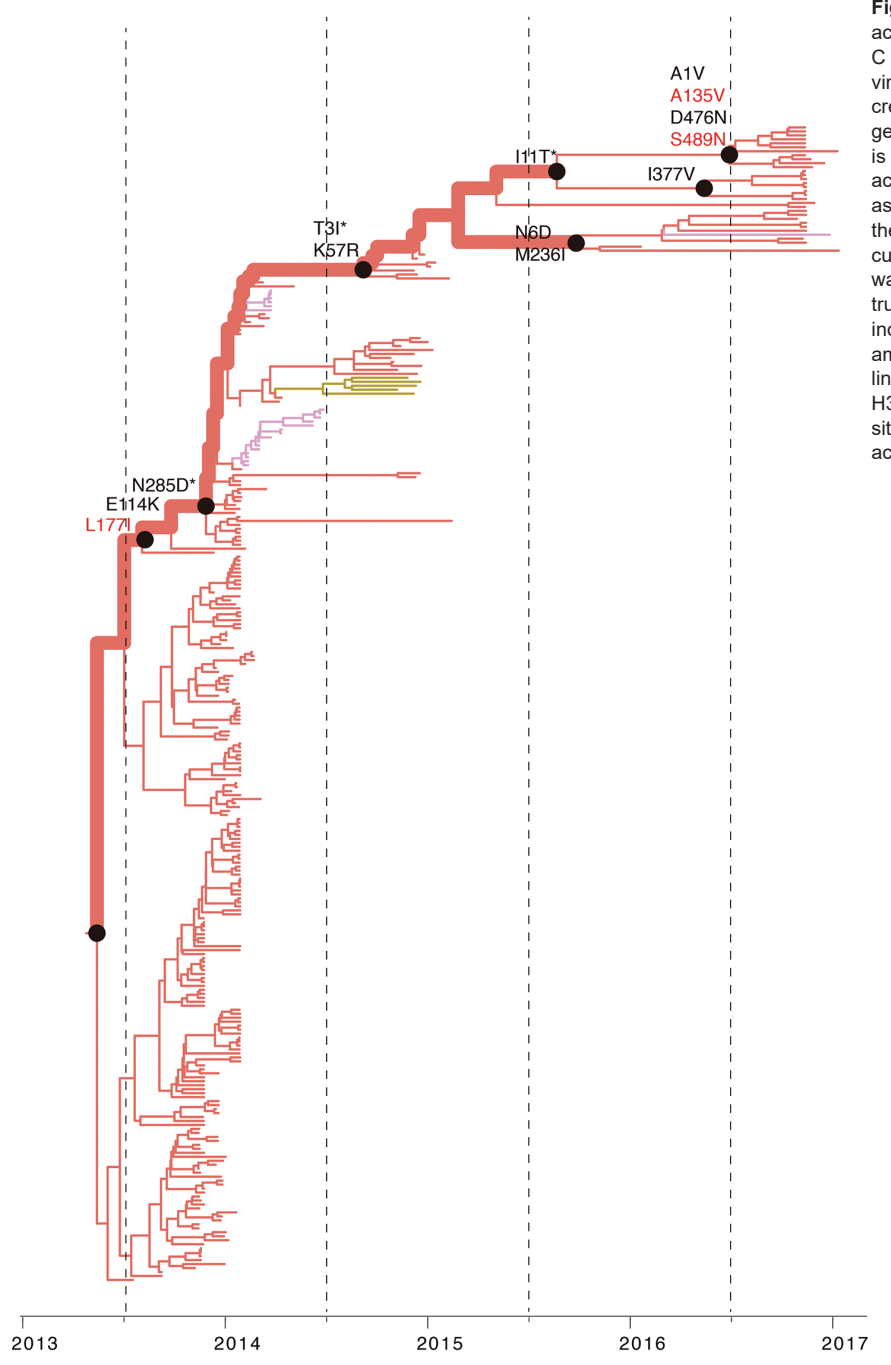
Reconstruction of amino acid changes along trunk of lineage C phylogenies of influenza A(H7N9) viruses, China. Maximum clade credibility tree of hemagglutinin gene sequences from lineage C is shown. Branches are colored according to geographic locations, as in Figure 3. Thicker lines indicate the trunk lineage leading up to the current fifth influenza epidemic wave. Amino acid changes along the trunk are indicated. Red branches indicate sites undergoing parallel amino acid changes across multiple lineages. Mutations correspond to H3 numbering scheme. *Mutation sites not present are numbered according to H7 numbering.

We then investigated whether the increasing prevalence of lineage C viruses might be associated with virus adaptation. We combined ancestral sequence reconstruction of lineage B and C HA gene sequences (Figures 4, 5) by mapping residues that have undergone changes onto the crystal structure of the trimeric hemagglutinin. Our analysis identified several notable amino acid substitutions that occurred along the internal branches of lineage C viruses (Figure 4).

**Figure 6:**
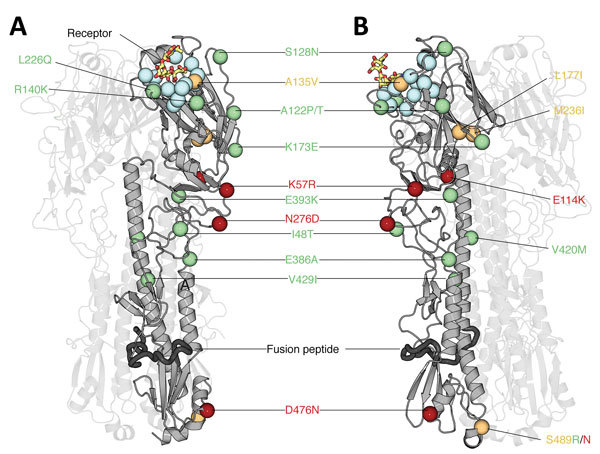
Structural analysis of amino acid changes in hemagglutinin in lineages B and C of influenza A(H7N9) viruses, China. Crystal structure of the homotrimeric H7 hemagglutinin bound to a human receptor analog (Protein Data Bank no. 4BSE) (*27*) (A) and rotated 90° counterclockwise (B) are shown. Two of the 3 protomers are displayed with high transparency to aid visualization. The carbon Cα positions of salient features are shown as spheres. Blue indicates receptor-binding residues, red indicates mutations in lineage B, green indicates mutations in lineage C, and orange indicates mutations in lineages B and C. Human receptor analog α2,6-SLN is shown as sticks colored according to constituent elements: carbon in orange, oxygen in red, and nitrogen in blue. Dark gray indicates the putative fusion peptide (*32*). Residues are numbered according to the H3 numbering system (Technical Appendix Table 2). A135 and L226 participate in receptor binding and thus are likely to modulate receptor specificity.

Around the time of the second influenza epidemic wave, ancestral viruses of lineage C acquired several amino acid changes in HA, specifically L177I, G386A, S489R, and S128N (based on H3 sequence numbering). Three of these mutations (G386A, S489R, and S128N) are located in solvent-accessible regions of HA (Figure 6; Technical Appendix Table 2). Furthermore, S128N was found within the 130 loop and is proximal to the receptor surface (distance ≈20 Å) (Figure 6).

We found by evolutionary analysis that several HA sites acquired amino acid mutations independently in different phylogenetic clades. First, 4 mutations (A135V, L177I, M236I, and S489N) occurred independently along the trunk lineages that gave rise to the current lineage B and C viruses (Figure 5). Three of these mutations (A135V, M236I, and S489N) were observed only in the fourth and fifth influenza epidemic waves of lineage B (Figure 5). Second, comparison of the C1 and C2 clades also identified parallel amino acid changes within lineage C (A122T/P and M236I) (Figure 4).

The observation of parallel amino acid changes along those particular lineages (Technical Appendix Tables 2, 3) that have persisted until the fifth influenza epidemic wave (i.e., parallel changes between lineages B and C and between the C1 and C2 clades) is suggestive of convergent, adaptive molecular evolution. The parallel changes in lineage C (A122T/P and M236I) are estimated to be fully or partially solvent accessible and the A135V mutation is located at the receptor-binding site (Figure 6). One subclade of lineage B viruses appears to have acquired mutations A135V and S489N within the last 12 months (Figure 5). Therefore, we suggest that this subclade should be closely monitored in the future.

Within the C2 clade, we found that HA acquired 7 amino acid changes (I48T, A122P, K173E, L226Q, M236I, I326V, and E393K) on the internal branch immediately ancestral to the HP cluster. This internal branch represents a period of approximately 1 year (Figure 4). Although all of these changes appeared in residues with partial or full solvent accessibility, mutations K173E, L226Q, and I326V are particularly noteworthy because they have arisen at or near known antigenic, receptor-binding, and proteolytic cleavage sites, respectively (Figure 6). Furthermore, these mutations in the HP cluster also coincide with appearance of a 4-amino acid insertion (KRTA) near the HA1-HA2 proteolytic cleavage site (Figure 4). A subset of HA substitutions (at sites 57, 114, 140, 226, and 276) that occurred on the trunk branches of lineages B and C viruses was also found to be under positive selection on the basis of dN/dS ratios we estimated by using methods implemented in HyPhy (Technical Appendix Table 4).

We also investigated whether amino acid changes in the HA gene during emergence of influenza A(H7N9) virus have been driven by adaptive evolution similar to that observed for seasonal human influenza (*30*). We found evidence for adaptive evolution in HA genes of B and C virus lineages. We estimated that lineage B adapted at a rate of 0.80 (interquartile range [IQR] 0.21–0.95) adaptive substitutions across the whole HA gene per year and lineage A at a rate of 0.60 (IQR 0.10–1.18) adaptive substitutions per year. Within lineage C, the estimated adaptation rate of the C1 clade is ≈1.84 (IQR 1.09–2.14) adaptive substitutions per year and that for the C2 clade (which includes the HP cluster) is 3.12 (IQR 2.45–3.79) adaptive substitutions per year. These results indicate molecular adaptation across the whole H7N9 lineage and suggest that adaptation is faster in the 2 C clades than in the A and B lineages. Previous estimates of rates of adaptive substitution were 1.02 fixations per year in the whole HA gene for seasonal human influenza A(H1N1) virus and 1.52 fixations per year in the whole HA gene for influenza A(H3N2) virus (*30*). In this context, the rate of adaptive evolution observed for lineage C here is notable and raises concern for ongoing evolution of these viruses.

### Antigenic Properties

We collected serum samples from 4 patients infected with H7N9 virus during 2015 and 2017 (Table). For patients 3 and 4, the corresponding virus strains were isolated and sequenced. Phylogenetic analysis indicated that patient 3 was infected with clade C1 virus and that patient 4 was infected with HP virus. Hemagglutination inhibition results suggested the presence of 3 antigenic clusters among the 16 H7N9 virus strains selected. Serum samples from patients 1, 2, and 3 showed similar patterns, reacting robustly to clade C1 viruses and moderately to clade C2 and lineage B viruses but poorly to HP viruses. A serum sample from a patient infected with an HP H7N9 virus appeared to react strongly to all H7N9 virus strains.

## Discussion

Our results show that H7N9 viruses of lineage C, which were prevalent in the recent fifth influenza epidemic wave in China, comprise 2 geographically distinct clades (C1 and C2) that have undergone adaptive evolution. Clade C1 is found primarily in eastern and central China and clade 2 in Guangdong Province, and both clades appear to have circulated in bird populations for ≈3 years. Our ancestral state reconstruction analysis provides crucial evidence that 2 successful lineages of H7N9 viruses (lineages B and C) have experienced multiple parallel amino acid changes (Figures 4, 5), suggesting the possible action of convergent molecular evolution.

We also observed a higher rate of virus adaptation in eastern Guangdong Province (C2 clade compared with C1). Although clades C1 and C2 are phylogenetically closely related, serum from a clade C1 virus-infected patient has moderate reactivity with C2 strains from 2015–2016 and poor reactivity to the HP virus from 2016–2017. The higher adaptation rate and antigenic changes in clade C2 are of concern from a public health perspective. Introduction of HP avian influenza into domestic poultry might constitute a serious risk, as demonstrated by emergence of goose–Guangdong lineage HP H5N1 viruses, which spilled back into wild birds and caused the longest global outbreak of HP avian influenza to date (*33*).

Parallel amino acid changes in clades C1 and C2 occurred at 2 sites in HA (122 and 236) (Figure 6). Furthermore, we observed 4 mutations that emerged independently in lineages B and C (sites 135, 177, 236, and 489). These results suggest adaptive convergent molecular evolution. Site 135 is located in the receptor-binding region and is near antigenic site A, as defined by Wiley et al. (*34*). Thus, the observed A135V mutation might modulate receptor affinity and contribute to immune escape (Figure 6), as observed in influenza A(H7N1) and A(H7N7) viruses (*35**,**36*).

Specifically, experimental studies indicate that threonine at position 135 in the HP H7N7 virus A/Netherlands/219/2003 confers broad-scale resistance to neutralizing monoclonal antibodies against the earliest strain of H7N9 virus (A/Shanghai/02/2013) (*37*). Furthermore, the World Health Organization has reported that recent clade C1 viruses (but not those of lineage B) react less to postinfection ferret antiserum raised against the A/Anhui/1/2013 and A/Shanghai/2/2013-derived candidate vaccine strains (*9*). Consistent with this finding, we found that most clade C1 viruses isolated in 2015 have the A135V mutation. However, this mutation was only detected in a small proportion of recent lineage B viruses (Figure 5).

In this study, we performed a preliminary evaluation of the antigenicity of H7N9 viruses by using patient serum samples collected in 2015 and 2017. Without serum raised in response to early strains from 2013, we cannot discriminate antigenic change between strains from 2013 and those from 2017. However, the limited data we have indicate the presence of 3 antigenic clusters among the 4 phylogenetic clusters circulating during the fifth influenza epidemic wave (Table). HA1 positions 109–301 (H3 numbering) include the A–E antigenic epitopes, which are known to determine antigenicity of influenza A viruses (*34*). The amino acid changes responsible for the antigenic differences between clade C1 and other clades were located in antigenic site A (position 140, H3 numbering; Technical Appendix Figure 2).

The mutation R140K has been observed in viruses isolated from ferrets experimentally infected with avian influenza A(H7N9) viruses and has been linked to antigenic drift of influenza A(H5N1) viruses (*38**–**40*). By comparing the sequences of the HP H7N9 virus cluster and other clade C2 viruses, we found a substitution in antigenic site E (position 173, H3 numbering) that could underlie antigenic change in HP H7N9 viruses (Technical Appendix Figure 2). In future work, we aim to explore the roles of these substitutions in determining viral antigenicity in the context of H7N9 virus genomes by using reverse genetics.

HP H7N9 viruses belonging to lineage C2 were first detected in late 2016, but spread greatly in geographic range in early 2017 (*41*). Several mechanisms for the genesis of an HP virus from a low pathogenicity virus have been proposed, including transcription errors (*42*), stepwise amino acid substitutions (*43*), or recombination (*44*). For H7, emergence of HP viruses is attributed to nonhomologous recombination resulting in simultaneous insertion of several amino acids at the HA cleavage site. These insertions might be derived from host 28S rRNA sequence (*45*) or from other influenza gene segments, such as the matrix (*46*) and nucleoprotein genes (*44*). The 12-nt insert in the HP H7N9 virus strains is 100% identical to a region in the polymerase basic 1 gene in multiple avian influenza A viruses, including subtypes H3N2, H6N2, and H9N2, but is not present in the polymerase basic 1 gene of HP H7N9 virus. H9N2 virus is the most frequently detected avian influenza virus in chickens in China, and the detection rate of this subtype in environmental samples from live poultry markets is ≈20% during the influenza epidemic season (*13*). Therefore, co-infection with H7N9 and other avian influenza viruses, such as influenza A(H9N2) viruses, could, in theory, lead to insertion of a polybasic cleavage site by nonhomologous recombination.

Recent studies have shown that the HP H7N9 virus is more pathogenic in mice, and more thermally stable, than low pathogenicity A/Anhui/1/2013 virus (*47**,**48*). Current surveillance indicates that HP H7N9 viruses have spread to several provinces in China and are responsible for large influenza outbreaks in poultry in central and northern China that show high mortality rates (http://www.fao.org/ag/againfo/programmes/en/empres/H7N9/situation_update.html). This finding raises the possibility of global dissemination of H7N9 viruses through migration of wild birds, in a manner similar to that observed for HP H5N1 viruses first identified in Guangdong Province (*32*). Although vaccination of poultry against H7N9 virus has now been implemented in some regions of China, virus adaptation and spatial distribution should be more closely monitored.

Technical Appendix Additional information on molecular evolution, diversity, and adaptation of influenza A(H7N9) viruses in China.
