# Supplementary material for: Molecular Evolution, Diversity, and Adaptation of Influenza A(H7N9) Viruses in China
Source: Emerg Infect Dis. 2018 Oct;24(10):1795–805. doi: 10.3201/eid2410.171063 (PMC6154164; doi:10.3201/eid2410.171063)
Supplement: Technical Appendix — Additional information on molecular evolution, diversity, and adaptation of influenza A(H7N9) viruses in China. [file 17-1063-Techapp-s1.pdf]

Article DOI: https://doi.org/10.3201/eid2410.171063

Molecular Evolution, Diversity, and Adaptation of Influenza A(H7N9) Viruses in China

Technical Appendix

Materials and Methods

Collection of Environmental Samples

As described (*1*–*3*), the Guangdong Provincial Center for Disease Control and Prevention launched an environmental surveillance program to monitor avian influenza viruses in live-poultry markets (LPMs) in 21 prefecture cities in April 2013. For each city, ≥1 local LPM was selected on the basis of their poultry sales and market coverage. A total of 625 LPMs were included in the environment surveillance study.

Because of seasonality in the prevalence of avian influenza, surveillance of LPMs was performed during November–May in 11 cities and each month in 10 other cities. At least 20 environmental samples were collected per market each week during the surveillance period. Environmental samples were collected as wet swab specimens from poultry feces, chicken epilator surfaces, chopping board surfaces, case surfaces, and sewage (*3*). If infection of a person with influenza A(H7N9) virus was confirmed and the person had exposure to an LPM, >20 environmental samples were collected from that market. The method of sample collection and detection used in this study has been described (*2*).

Collection of Serum Samples

Serum samples were collected from 4 patients infected with influenza A(H7N9) virus: 2 (patients P1 and P2) in 2015 and 2 (patients P3 and P4) in 2017 (Table). Respiratory specimens were collected for reverse transcription PCR and blindly passaged for 2–3 generations in embryonated chicken eggs for virus isolation. Serum samples were collected 2–3 weeks after clinical symptoms were observed and were aliquoted and stored at −70°C. H7N9 subtype virus strains (EPI1171790/A/ZS29 and EPI1171792/A/ST18) were isolated from respiratory specimens of P3 and P4 (Table).

Hemagglutination Inhibition Assay

Assays were performed according to the laboratory procedure defined by the World Health Organization (

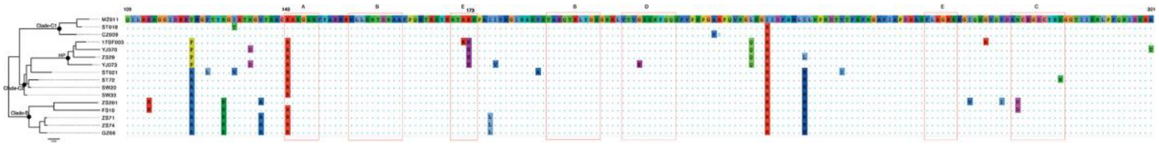

http://www.who.int/influenza/gisrs_laboratory/cnic_serological_diagnosis_hai_a_h7n9_20131220.pdf). For H7N9 subtype virus, horse erythrocytes were used in the assay to achieve improved sensitivity. To exclude nonspecific agglutination, patient serum was treated by using hemadsorption with horse erythrocytes and then receptor-destroying enzyme treatment. In brief, 100 μL of original serum was first heat-inactivated at 56°C for 30 min and then diluted with 400 μL of phosphate-buffered saline. A total of 50 μL of packed horse erythrocytes was then added, and the sample was incubated for 15 min at 4°C for adsorption. After centrifugation, ≈400 μL of diluted serum was recovered and treated with 1.2 mL of receptor-destroying enzyme at 37°C overnight. Treated serum samples were aliquoted and stored at –70°C until used.

Hemagglutinin titers for influenza A(H7N9) virus reference strains from each clade (Table) were first determined by using hemagglutination testing. Samples were then diluted to make a working solution containing 8 hemagglutinating units/50 µL. Two-fold serial dilutions of treated serum samples (25 µL) were subsequently prepared and mixed with 25 µL of standardized virus containing 4 hemagglutinating units. Mixtures were incubated for 30 min at room temperature before hemagglutination testing. Virus back titration was performed in parallel. Each experiment was repeated twice, and HI titers (Table) shows mean values from the 2 experiments.

References

1. Lu J, Wu J, Zeng X, Guan D, Zou L, Yi L, et al. Continuing reassortment leads to the genetic diversity of influenza virus H7N9 in Guangdong, China. J Virol. 2014;88:8297–306. PubMed http://dx.doi.org/10.1128/JVI.00630-14

2. Ke C, Lu J, Wu J, Guan D, Zou L, Song T, et al. Circulation of reassortant influenza A(H7N9) viruses in poultry and humans, Guangdong Province, China, 2013. Emerg Infect Dis. 2014;20:2034–40. PubMed http://dx.doi.org/10.3201/eid2012.140765

3. Kang M, He J, Song T, Rutherford S, Wu J, Lin J, et al. Environmental sampling for avian influenza A(H7N9) in live-poultry markets in Guangdong, China. PLoS One. 2015;10:e0126335. PubMed http://dx.doi.org/10.1371/journal.pone.0126335

4. Gouet P, Courcelle E, Stuart DI, Métoz F. ESPript: analysis of multiple sequence alignments in PostScript. Bioinformatics. 1999;15:305–8. PubMed http://dx.doi.org/10.1093/bioinformatics/15.4.305

5. Winn MD, Ballard CC, Cowtan KD, Dodson EJ, Emsley P, Evans PR, et al. Overview of the CCP4 suite and current developments. Acta Crystallogr D Biol Crystallogr. 2011;67:235–42. PubMed http://dx.doi.org/10.1107/S0907444910045749

6. Wiley DC, Wilson IA, Skehel JJ. Structural identification of the antibody-binding sites of Hong Kong influenza haemagglutinin and their involvement in antigenic variation. Nature. 1981;289:373–8. PubMed http://dx.doi.org/10.1038/289373a0

Technical Appendix Table 1. Geographic distribution of human case of infection with influenza A(H7N9) virus (as of April 8, 2017) and HA and NA gene sequences used in phylogenetic analyses, China*

Location in China	No. clinical cases	No. HA sequences	No. NA sequences
Eastern	709	271	210
Central	134	66	77
Northern	47	12	15
Southeastern	105	26	31
Central Guangdong	211	296	219
Eastern Guangdong	61	66	58

*HA, hemagglutinin; NA, neuraminidase.

Technical Appendix Table 2. Amino acid substitutions (H3 numbering) identified on trunk branches of H7 gene of influenza A(H7N9) viruses, China*

Mutation	Associated lineage	Solvent accessibility†	Salient feature
HA1			
A1V	B	Unknown	NA
N6D	B	Unknown	NA
I48T	C2	Partial	NA
K57R	B	Full	NA
E114K	B	Partial	NA
A122P/T	**C1, C2**	Full	NA
S128N	C	Full	Within 130 loop
A135V	B, C	Full	Receptor binding residue‡
R140K	C1	Full	Antigenic site A§
K173E	C2	Full	Antigenic site E§
L177I	B, C	Inaccessible	Antigenic site E§
L226Q	C2	Partial	Receptor binding residue‡
M236I	**C1, C2**	Partial	NA
N276D	B	Full	Antigenic site C§
I326V	C2	Unknown	Proteolytic cleavage site
HA2			
E386A	C	Full	NA
E393K	C2	Partial	NA
V429I	C1	Inaccessible	NA
D476N	B	Full	NA
S489R/N	B, C	Full	NA

*Independent mutations on two or more lineages are highlighted in bold. HA, hemagglutinin; NA, not applicable.
†As defined by analysis with ESPript (*4*) using the biologic assembly of PDB: 4BSE.
‡Receptor-binding residues were determined by using CONTACT in CCP4 (*5*).
§Antigenic sites as defined by Wiley et al. (*6*).

Technical Appendix Table 3. Amino acid substitutions (N2 numbering) on trunk branches of the N9 gene of avian influenza A(H7N9) viruses, China*

Mutation	Associated Lineage
I16T	**B, C1, C2**
G18S	B
A21T	C2
V22A	B
H55R	C2
T60I	B
M82I	C1
S45P	C1
T181A	C1
V205I	C2
V212I	C1
S245P	**C1, C2**
E283K	C2
I303V	C1
V308I	B
N325S	**C1, C2**
N347S	B
V349I	B
A358T/D	**C1, C2**
R430K	C2

*Independent mutations for ≥2 lineages are indicated in bold.

Technical Appendix Table 4. Putative sites (H3 numbering) that undergo positive selection in influenza A(H7N9) virus lineages B and C (clades C1 and C2), China*

	Method			
Lineage	SLAC	FEL	MEME	FUBAR
B	None	57, 140	57, 140	57, 140, 276, 291, 493
C1	None	57, 424	57, 424	57, 114, 424
C2	226	226	3, 226, 486	226, 332

*FEL, fixed-effects likelihood; FUBAR, fast unconstrained Bayesian approximation; MEME, mixed-effects model of evolution; SLAC, single-likelihood ancestor counting.

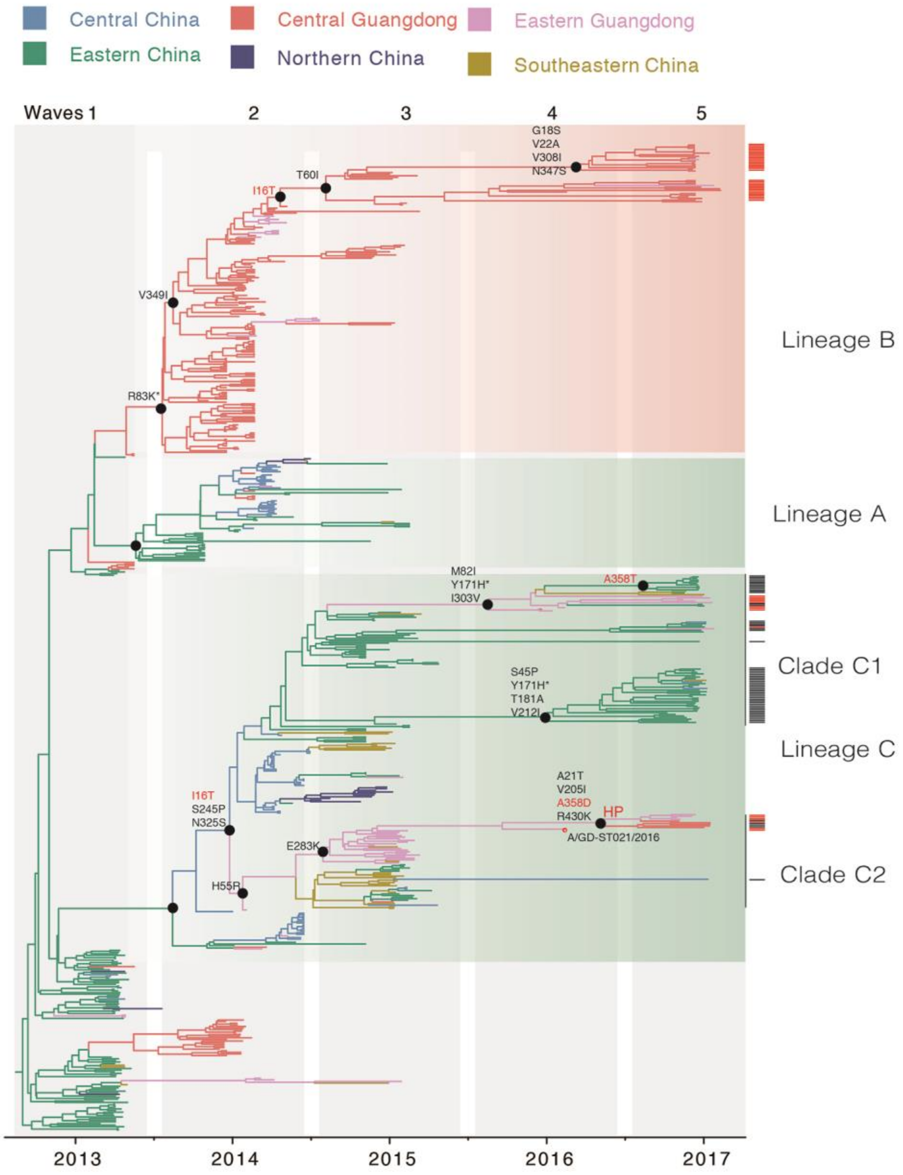

Technical Appendix Figure 1. Bayesian maximum clade credibility tree of neuraminidase genes from influenza A(H7N9) viruses, China. Published sequences (from waves 4 and 5) from other studies are indicated by black bars to the right of the tree, and sequences reported in this study from Guangdong are indicated by red bars. Branches are colored according to geographic locations, as in Figure 1. Amino acid changes are mapped on trunk branches of B and C lineages. Parallel changes that occur in both lineages are indicated in red. Mutations are

numbered according to the N2 scheme, with the exception of mutations marked by *, which are not present in the N2 protein and are based on the N9 numbering scheme.

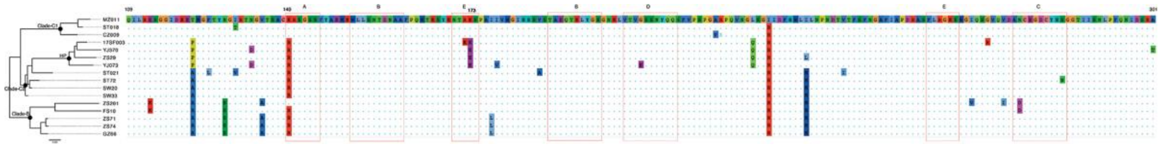

Technical Appendix Figure 2. Sequence alignment of hemagglutinin genes of influenza A(H7N9) virus strains used in the hemagglutination inhibition test in Technical Appendix Table 1, China. The region (109–301, H3 numbering) determining viral antigenicity is shown, and A–E antigenic epitopes are indicated by red boxes. Different colors indicate different amino acids. Dots indicate sequence identity. Scale bar indicates amino acid substitutions per site.
